# Effect of changes in Breslow thickness between the initial punch biopsy results and final pathology reports in acral lentiginous melanoma patients

**DOI:** 10.1038/s41598-021-99422-6

**Published:** 2021-10-06

**Authors:** Tae Hyung Kim, Jin Cheol Kim, Ji Eun Kwon, You Chan Kim, Jee Woong Choi

**Affiliations:** 1 Department of Dermatology, Ajou University School of Medicine, Ajou University Hospital, 164, World Cup-ro, Yeongtong-gu, Suwon-si, Gyeonggi-do, Suwon, 16499 South Korea; 2 Department of Pathology, Ajou University School of Medicine, Suwon, South Korea

**Keywords:** Cancer, Risk factors

## Abstract

Acral lentiginous melanoma (ALM) is the most common subtype of cutaneous melanoma among Asians; punch biopsy is widely performed for its diagnosis. However, the pathologic parameters evaluated via punch biopsy may not be sufficient for predicting disease prognosis compared to the parameters evaluated via excisional biopsy. We investigated whether changes in Breslow thickness (BT) between initial punch biopsy results and final pathology reports can affect the prognosis of ALM. Pathologic parameters were recorded from specimens acquired through the initial punch biopsy and wide excision. Patients were classified into two groups based on a change in Breslow depth: the BT increased or decreased on comparing the samples from the initial punch biopsy and final wide excision. We compared clinical characteristics, and a Cox regression model was used to identify independent prognostic factors influencing melanoma-specific death (MSD). Changes in BT did not affect MSD (hazard ratio [HR]: 0.55, *P* = 0.447). In multivariate analysis, a higher BT (> 2 mm) (HR: 9.93, *P* = 0.046) and nodal metastasis (HR: 5.66, *P* = 0.041) were significantly associated with an increased MSD risk. The use of punch biopsy did not affect MSD despite the inaccuracy of BT measurement as long as ALM was accurately diagnosed.

## Introduction

Acral lentiginous melanoma (ALM) is a subtype of cutaneous melanoma that has a worse prognosis than the other melanoma subtypes^1^. Although the absolute incidence of cutaneous melanoma is generally low among Asians, ALM is the most common subtype in this population; ALM accounts for 47%–86.6% of cutaneous melanoma cases^2^.

The poor prognosis of ALM is attributed to delayed diagnosis, which contributes to an increase in tumor thickness^3^. Teramoto et al.^2^ reported that four parameters, namely age, ulceration, Breslow thickness (BT), and tumor spread, were independent significant prognostic factors for disease-specific survival in ALM patients.

ALM can be misdiagnosed as nevi or other benign conditions, making early diagnosis difficult^4–6^. Because delayed diagnosis allows the tumor to get thicker and hence affect prognosis, early diagnosis is important. Moreover, many studies have reported that increased BT is associated with lower melanoma-specific survival rates and higher melanoma recurrence rates^3,7–9^.

Skin biopsy is an important step for the confirmative diagnosis of melanoma and may be performed by removing a part of a lesion or the entire lesion. In general, excisional biopsy is strongly recommended when the melanoma is clinically suspected to be representative of the exact BT. Moreover, an excisional biopsy can encompass a sufficient depth that would prevent transection at the base of the lesion. However, this method may not be suitable in certain circumstances: for instance, when the tumor is too large to be primarily excised or is located in cosmetically or functionally sensitive areas such as the palm, sole, or distal digits^10–13^. In these scenarios, a partial biopsy method such as punch biopsy is widely used when melanoma is suspected; however, it may not accurately reflect the BT of the tumor. Therefore, clinicians can be faced with inaccurate surgical decision-making and prognosis based on incomplete histologic information^11^.

In this study, we investigated whether changes in BT between the initial punch biopsy results and final pathology reports affected the prognosis of ALM patients.

## Results

The clinical characteristics and histological information for groups 1 and 2 are shown in Table 1. In total, 18 and 26 patients were classified in group 1 and group 2, respectively. The median of the final BT was 2.1 mm in group 1 and 2.9 mm in group 2. Other characteristics such as ulceration, mitosis rate, microsatellites, and lymphovascular invasion were observed more frequently in group 2; however, there was no significant difference between the groups. The mean overall follow-up period was 4.62 years; the mean follow-up periods in group 1 and group 2 were 4.82 and 4.49 years, respectively.

**Table 1:** Comparison of patient, clinical, and tumor characteristics between the two groups.

Characteristics	Total (N = 44)	Breslow thickness		*P* value
		Group 1 (N = 18)	Group 2 (N = 26)	
Patient characteristics				
Gender, n (%)				0.548
Men	17 (38.6)	6 (33.3)	11 (42.3)	
Women	27 (61.4)	12 (66.7)	15 (57.7)	
Age, year, mean ± SD	62.9 ± 11.3	61.0 ± 10.4	63.8 ± 11.9	0.411
Comorbidities, n (%)				
Hypertension	15 (34.1)	6 (33.3)	9 (34.6)	0.930
Diabetes	9 (20.5)	4 (22.2)	5 (19.2)	1.000
Dyslipidemia	2 (4.5)	1 (5.6)	1 (3.8)	1.000
Skin cancer	0 (0.0)	0 (0.0)	0 (0.0)	NA
Other internal cancer	8 (26.3)	3 (16.7)	5 (19.2)	1.000
Clinical characteristics				
SLNB performed, n (%)	28 (63.6)	14 (77.8)	14 (53.8)	0.125
^a^Nodal metastasis, n (%)	8 (18.2)	3 (16.7)	5 (19.2)	1.000
Surgery, n (%)				0.395
Wide excision	26 (59.1)	12 (66.7)	14 (53.8)	
Amputation	18 (40.9)	6 (33.3)	12 (46.2)	
Tumor characteristics				
Final Breslow thickness, mm				0.145
Median	2.5	2.1	2.9	
IQR	3.1	2.5	3.4	
Ulceration	16 (36.4)	5 (27.8)	11 (42.3)	0.325
Mitosis, n/mm^2^, n (%)				0.387
0–1	21 (47.7)	10 (55.6)	11 (42.3)	
2 <	23 (52.3)	8 (44.4)	15 (57.7)	
Microsatellite, n (%)	4 (9.1)	1 (5.6)	3 (11.5)	0.634
Lymphovascular invasion, n (%)	8 (18.2)	3 (16.7)	5 (19.2)	1.000
Regression, n (%)	6 (13.6)	3 (16.7)	3 (11.5)	0.676
Tumor infiltrating lymphocyte, n (%)				0.831
None	17 (38.6)	6 (33.3)	11 (42.3)	
Non-brisk	16 (36.4)	7 (38.9)	9 (34.6)	
Brisk	11 (25.0)	5 (27.8)	6 (23.1)	
Follow-up time per subject, year, mean ± SD	4.62 ± 3.77	4.82 ± 4.16	4.49 ± 3.56	0.778

*IQR* interquartile range, *NA* not applicable, *SLNB* sentinel lymph node biopsy, *SD* standard deviation.

^a^Clinically occult or detected lymph nodes.

Among all covariates presented in Table 1, the variables with *P*-values < 0.1 in univariate Cox analysis, except for changes in BT, are listed in Table 2. Group 2 had an increased risk of MSD; however, there was no statistical significance on comparison with group 1 (HR: 1.30, 95% CI: 0.38–4.22, *P* = 0.993). However, nodal metastasis (HR: 6.66, 95% CI: 2.01–22.08, *P* = 0.002) and a higher BT (> 2 mm) (HR: 7.62, 95% CI: 1.59–36.55, *P* = 0.011) were significantly associated with an increased risk of MSD.

**Table 2:** Cox models for melanoma-specific survival in relation to changes in Breslow thickness between the initial punch biopsy and final excision specimens.

	Univariate		Multivariate^a^	
	HR (95% CI)	*P*-value	HR (95% CI)	*P*-value
Breslow thickness change				
Decreased or same (group 1)	(Reference)		(Reference)	
Increased (group 2)	1.30 (0.38–4.22)	0.993	0.55 (0.12–2.53)	0.447
Nodal metastasis				
No	(Reference)		(Reference)	
Yes	6.66 (2.01–22.08)	0.002	5.66 (1.08–29.76)	0.041
Final Breslow thickness, mm				
≤ 2	(Reference)		(Reference)	
> 2	7.62 (1.59–36.55)	0.011	9.93 (1.04–94.96)	0.046
Ulceration				
No	(Reference)		(Reference)	
Yes	2.76 (0.85–8.92)	0.090	2.83 (0.63–12.71)	0.175
Mitosis, n/mm^2^				
0–1	(Reference)		(Reference)	
2 <	3.07 (0.88–10.64)	0.078	0.85 (0.15–4.91)	0.851
Lymphovascular invasion				
No	(Reference)		(Reference)	
Yes	2.94 (0.87–9.90)	0.082	4.04 (0.88–18.47)	0.072

*CI* confidence interval, *HR* hazard ratio.

Among all covariates in Table 1, variables with *P*-values less than 0.1 in univariate Cox analysis were listed (except for changes in Breslow thickness) and included (with age and sex) in multivariate analysis.

^a^Multivariate Cox analysis was performed using age, sex, and all variables in the list.

**Figure 1:**
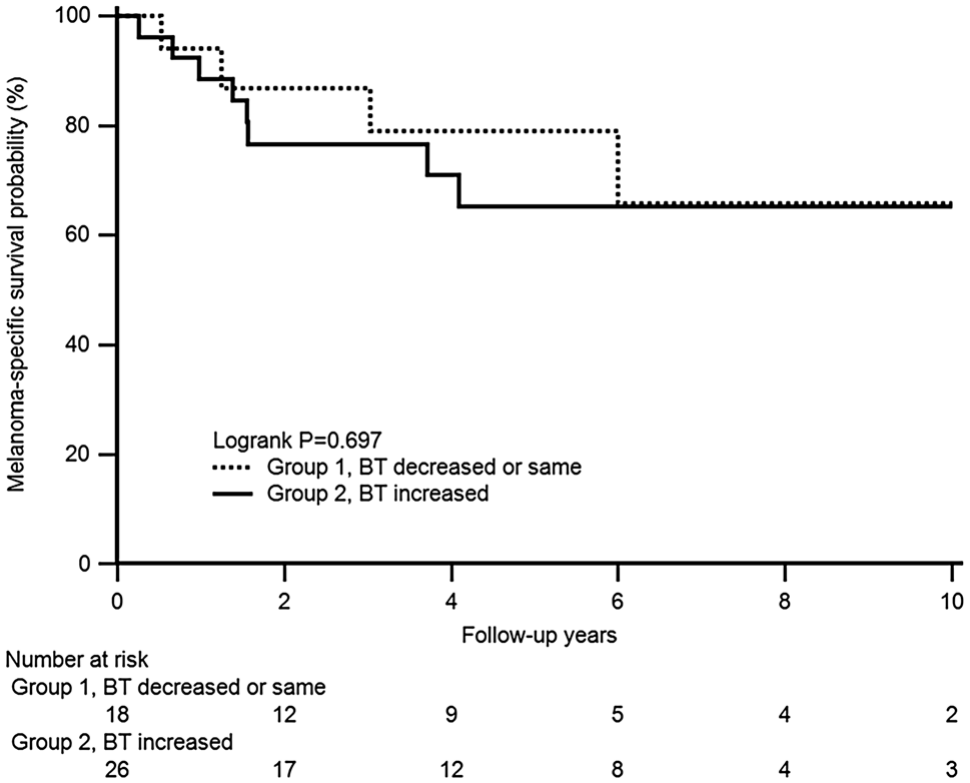
Comparison of the melanoma-specific survival probability between the two groups.

According to the multivariate analysis including age, sex, and the covariates presented in Table 2, group 2 did not show a significantly increased risk of MSD compared to group 1 (HR: 0.55, 95% CI: 0.12–2.53, *P* = 0.447). However, a higher BT (HR: 9.93, 95% CI: 1.04–94.96, *P* = 0.046) and nodal metastasis (HR: 5.66, 95% CI: 1.08–29.76, *P* = 0.041) were still significant risk factors of MSD. In the comparison of the melanoma-specific survival rate between group 1 and group 2, no significant differences were observed (log-rank *P* = 0.697) (Fig. 1).

## Discussion

In this study, we compared the ALM survival rate according to changes in BT between the initial punch biopsy and wide excision samples from an Asian population. Although punch biopsy is a useful and safe method to diagnose ALM without removing the entire tumor and although there is no definite evidence that it increases the risk of tumor seeding into the cutaneous lymphatics or blood vessels^14^, this technique has the disadvantage of underestimating the final BT, which is the most important factor affecting the prognosis of melanoma^2,3,7,8^. Nevertheless, our study revealed that changes in the initial and final BTs did not affect MSD among our patients and that the final BT and nodal metastasis were significantly associated with MSD. In other words, even if the exact BT is not estimated through punch biopsy, the survival rate of ALM patients remains unaffected.

When partial biopsy (e.g., punch and shave) is used, every 1 mm increase in tumor thickness causes an increase in microstaging inaccuracy (vs. final excision; odds ratio: 1.8; *P* < 0.001)^15^. A previous study showed that about 5% of partial biopsies, such as punch and shave biopsies, in the cutaneous melanoma demonstrated sufficient discrepancy for changing surgical recommendations^16^. Despite these disadvantages, Molenkamp et al.^17^ showed that there was no significant relationship between biopsy type and survival through univariate and multivariate testing. Moreover, Mills et al.^18^ reported that punch biopsy did not affect the accuracy of sentinel lymph node biopsy, tumor recurrence, and disease-specific survival but reported that it could lead to tumor upstaging. We obtained results consistent with these findings among our patients with ALM.

Our study has several limitations. First, this study did not perform a direct comparison between a punch biopsy and excisional biopsy. It is not easy to directly compare these two techniques because excisional biopsy is rarely performed in the clinical setting for ALM. Second, our study had a relatively small number of patients and was a retrospective study conducted in a single tertiary center. To evaluate the survival rate more accurately, a large-scale, multi-center study is required. Third, punch biopsy alone may not accurately diagnose ALM or ALM in situ because it removes part of the lesion, missing the diagnosis of melanoma in 7% cases of dysplastic nevus^19^. However, given the characteristics of ALM with a long radial growth phase, when ALM is strongly suspected, this disadvantage can be overcome through continuous follow-up and rebiopsy^20^. Moreover, using a dermatoscope to increase the accuracy of diagnosis can be a useful alternative^21,22^.

In conclusion, we confirmed that the final BT and nodal metastasis were significant factors affecting the survival rate in ALM patients. We also revealed that changes in BT did not affect the survival rate in ALM patients, even though BT may not be accurately measured through punch biopsy. Therefore, punch biopsy can be a useful modality for the diagnosis of ALM or ALM in situ that does not affect the disease prognosis.

## Materials and methods

### Study participants

**Figure 2:**
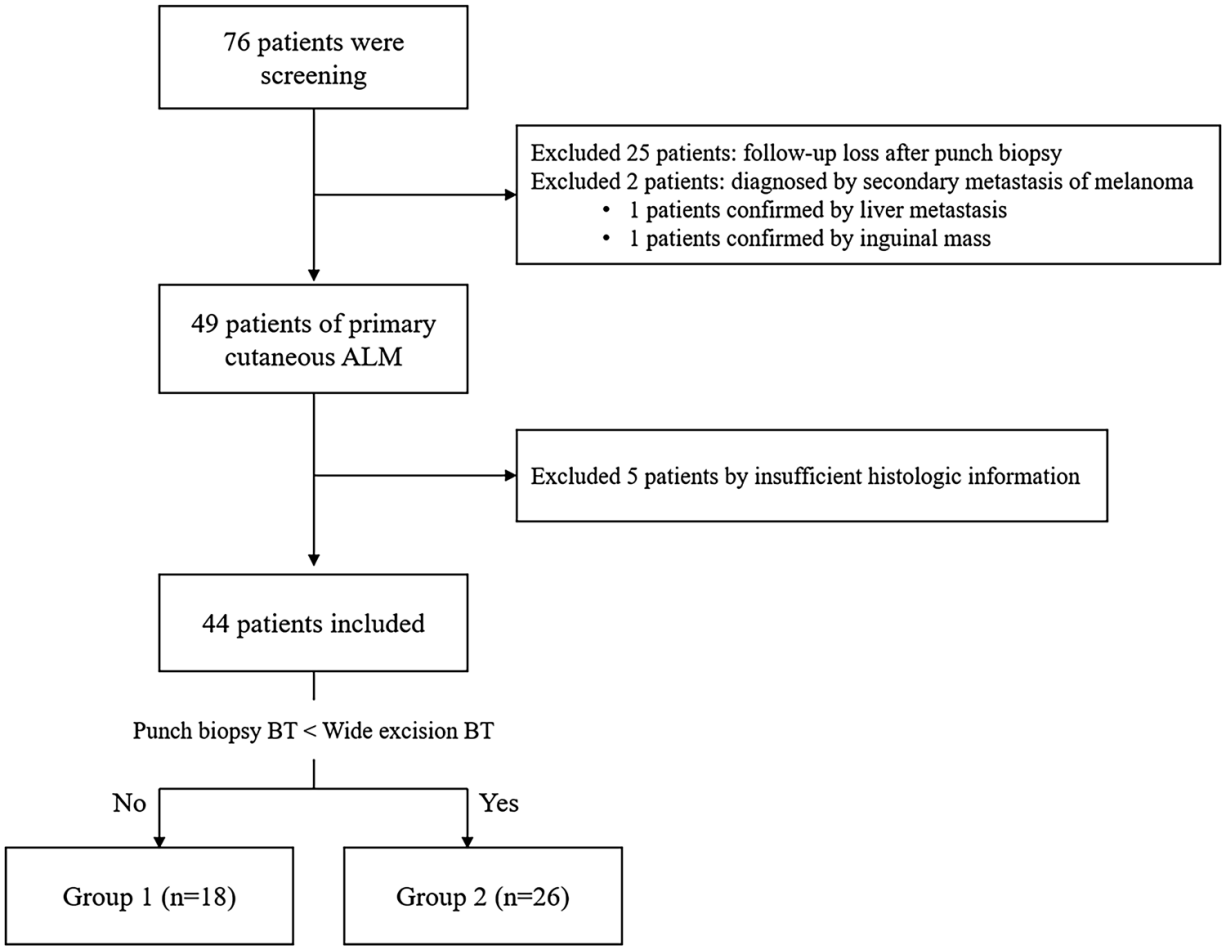
Flowchart describing our study population. *BT* Breslow thickness.

We retrospectively reviewed the medical records of patients diagnosed with ALM between January 2003 and December 2020 in the Department of Dermatology of Ajou University Hospital, Korea. The inclusion criteria were as follows: (1) diagnosis of primary cutaneous ALM or ALM in situ through initial punch biopsy; (2) availability of both initial punch biopsy and subsequent wide excision samples; and (3) availability of information on all pathologic parameters, as recorded by dermatopathologists. A total of 76 patients were screened. The 32 patients who did not meet the inclusion criteria were excluded, and 44 patients were enrolled in the study (Fig. 2).

### Data collection

Data on the demographics and clinical characteristics of the study participants were collected by reviewing electronic medical records. We collected data on pathologic parameters such as BT (mm), ulceration, mitotic rate (per mm^2^), microsatellites, lymphovascular invasion, regression, and tumor infiltrating lymphocytes (brisk, non-brisk, and absent) from the initial punch biopsy and wide excision specimens.

After comparing the BT of the initial punch biopsy specimens with that of the wide excision specimens in the same patients, the higher value was considered as the final BT. For the other pathologic parameters, the worse pathologic findings between the initial punch biopsy and wide excision specimens were defined as the final pathologic parameters. To evaluate the survival rate among patients based on changes in BT, patients were classified into two groups based on a change in Breslow depth: the thickness was increased or decreased on comparing samples from the initial punch biopsy and final wide excision. In group 1, the BT of a punch biopsy specimen was equal to or greater than that of a wide excision specimen, and in group 2, the BT of a punch biopsy specimen was less than that of a wide excision specimen.

### Statistical analyses

Descriptive analyses including the χ^2^ test or Fisher’s exact test for categorical data and the t-test or non-parametric tests for numerical data were carried out to evaluate statistical significance. The hazard ratios (HRs) with 95% confidence intervals (CIs) of overall death and melanoma-specific death (MSD) in the patient group were estimated using Cox regression analysis. The Kaplan–Meier method was used to draw survival curves, and the log-rank test was used to assess the significance of differences. All statistical analyses were performed using SPSS software (IBM SPSS Statistics for Windows, Version 25.0. Armonk, NY).

### Ethics declarations

This study did not have a risk to access identifiable information of subjects. This study was approved and written informed consent was waived by the institutional review board of Ajou University Hospital (AJIRB-MED-MDB-19-536). All researchers conducted this study according to relevant guidelines and regulations.
